# Supplementary material for: Widespread male sterility and trioecy in androdioecious Mercurialis annua: Its distribution, genetic basis, and estimates of morph‐specific fitness components
Source: Am J Bot. 2024 Oct 31;111(11):e16429. doi: 10.1002/ajb2.16429 (PMC11584041; doi:10.1002/ajb2.16429)
Supplement: Supplementary file 1 — Appendix S1. Coordinates, numbers of sexual phenotypes, and density of 109 Mercurialis annua populations surveyed along a transect in Spain. [file AJB2-111-e16429-s001.docx]

**Appendix S1**. Coordinates, numbers of sexual phenotypes, and density of 109 *Mercurialis annua* populations transected in Spain.

ID: unique for each population

Herma: number of hermaphrodites on the surface surveyed

Female: number of females on the surface surveyed

Male: number of males on the surface surveyed

Neuter: number of male sterile males on the surface surveyed

Total: total number of individuals on the surface surveyed

Density: density of the surface surveyed

Long: longitude

Lat: latitude

| ID | Herma | Female | Male | Neuter | Total | Density | Long | Lat |
| --- | --- | --- | --- | --- | --- | --- | --- | --- |
| 29 | 37 | 54 | 53 | 0 | 144 | 72 | -6.337178 | 37.832464 |
| 19 | 98 | 2 | 54 | 0 | 154 | 154 | -6.25595 | 37.396689 |
| 78 | 45 | 32 | 39 | 0 | 116 | 29 | -0.157205 | 38.946296 |
| 49 | 59 | 10 | 33 | 0 | 102 | 68 | -8.59899 | 40.012432 |
| 46 | 120 | 1 | 56 | 0 | 177 | 177 | -8.683316 | 39.221228 |
| 15 | 83 | 3 | 37 | 0 | 123 | 82 | -5.900697 | 37.129764 |
| 16 | 111 | 7 | 50 | 0 | 168 | 56 | -6.239001 | 37.299954 |
| 30 | 55 | 35 | 38 | 0 | 128 | 42.7 | -6.337178 | 37.832464 |
| 60 | 84 | 0 | 34 | 0 | 118 | 29.5 | -8.63868 | 42.35668 |
| 79 | 43 | 33 | 30 | 0 | 106 | 53 | -0.164833 | 38.950063 |
| 21 | 92 | 0 | 36 | 0 | 128 | 64 | -5.814909 | 37.620779 |
| 40 | 93 | 18 | 41 | 0 | 152 | 76 | -8.594411 | 38.477326 |
| 75 | 53 | 34 | 31 | 1 | 118 | 11.8 | -0.211241 | 39.01596 |
| 101 | 179 | 40 | 70 | 3 | 289 | 145 | -4.802458 | 36.513278 |
| 76 | 108 | 32 | 40 | 0 | 180 | 45 | -0.161754 | 38.98614 |
| 97 | 179 | 18 | 55 | 1 | 252 | 42 | -5.23459 | 36.376288 |
| 36 | 143 | 6 | 41 | 0 | 190 | 190 | -8.634128 | 37.299928 |
| 18 | 71 | 26 | 26 | 0 | 123 | 20.5 | -6.265057 | 37.51376 |
| 11 | 83 | 14 | 26 | 0 | 123 | 30.8 | -5.961272 | 36.258628 |
| 114 | 89 | 0 | 22 | 2 | 111 | 111 | -4.012034 | 36.745179 |
| 56 | 82 | 0 | 18 | 0 | 100 | 66.7 | -8.644923 | 42.024842 |
| 33 | 344 | 12 | 77 | 0 | 433 | 433 | -7.484117 | 37.198668 |
| 26 | 98 | 1 | 19 | 0 | 118 | 59 | -5.239896 | 37.531955 |
| 89 | 105 | 0 | 20 | 0 | 125 | 52.1 | -3.911049 | 36.743326 |
| 37 | 114 | 10 | 22 | 0 | 146 | 20.9 | -8.647156 | 37.597748 |
| 9 | 115 | 0 | 20 | 0 | 135 | 33.8 | -5.600616 | 36.014828 |
| 6 | 117 | 0 | 20 | 0 | 137 | 34.3 | -1.495681 | 37.765168 |
| 80 | 83 | 5 | 15 | 0 | 103 | 147 | -0.196492 | 38.916637 |
| 112 | 77 | 18 | 15 | 1 | 110 | 27.5 | -4.094362 | 36.759159 |
| 77 | 93 | 6 | 15 | 0 | 114 | 11.4 | -0.144821 | 38.95441 |
| 34 | 108 | 0 | 14 | 0 | 122 | 163 | -8.026348 | 37.131445 |
| 35 | 170 | 0 | 21 | 0 | 191 | 95.5 | -8.566675 | 37.2915 |
| 90 | 130 | 0 | 16 | 0 | 146 | 9.13 | -3.798892 | 36.747653 |
| 1 | 156 | 4 | 18 | 0 | 178 | 8.9 | -2.972926 | 36.75812 |
| 28 | 153 | 1 | 17 | 0 | 171 | 171 | -6.170429 | 37.716344 |
| 111 | 91 | 0 | 9 | 0 | 100 | 100 | -4.106022 | 36.740644 |
| 92 | 75 | 17 | 8 | 2 | 100 | 100 | -3.39585 | 36.707991 |
| 99 | 125 | 26 | 13 | 0 | 164 | 16.4 | -5.281729 | 36.278229 |
| 13 | 158 | 12 | 14 | 0 | 184 | 20.4 | -6.212425 | 36.621983 |
| 102 | 97 | 0 | 7 | 0 | 104 | 26 | -4.50768 | 36.638679 |
| 12 | 117 | 10 | 9 | 0 | 136 | 22.7 | -6.052972 | 36.226171 |
| 98 | 96 | 22 | 8 | 2 | 126 | 16.8 | -5.444736 | 36.10137 |
| 8 | 104 | 0 | 7 | 0 | 111 | 55.5 | -5.577171 | 36.032888 |
| 104 | 96 | 14 | 7 | 0 | 117 | 117 | -4.436443 | 36.752844 |
| 108 | 127 | 19 | 9 | 0 | 155 | 77.5 | -4.354438 | 36.73025 |
| 74 | 89 | 12 | 6 | 0 | 107 | 10.7 | -0.554755 | 39.431718 |
| 50 | 101 | 0 | 6 | 0 | 107 | 107 | -8.476339 | 40.371306 |
| 105 | 201 | 8 | 12 | 0 | 221 | 44.2 | -4.426776 | 36.758724 |
| 106 | 115 | 7 | 7 | 0 | 129 | 129 | -4.424458 | 36.765204 |
| 116 | 79 | 28 | 6 | 0 | 113 | 113 | -3.584218 | 36.74292 |
| 38 | 180 | 0 | 10 | 0 | 190 | 190 | -8.714075 | 38.026941 |
| 14 | 179 | 9 | 10 | 0 | 198 | 79.2 | -5.831903 | 37.009411 |
| 110 | 91 | 6 | 5 | 0 | 102 | 68 | -4.251222 | 36.717739 |
| 93 | 97 | 5 | 5 | 0 | 107 | 7.93 | -4.648232 | 36.508862 |
| 23 | 144 | 0 | 7 | 0 | 151 | 37.8 | -5.083004 | 37.5294 |
| 109 | 129 | 2 | 6 | 0 | 137 | 91.3 | -4.267112 | 36.72005 |
| 27 | 91 | 5 | 4 | 0 | 100 | 16.7 | -5.628882 | 37.469369 |
| 3 | 168 | 0 | 7 | 0 | 175 | 77.8 | -2.395353 | 36.842598 |
| 113 | 100 | 2 | 4 | 0 | 106 | 70.7 | -4.059604 | 36.757848 |
| 7 | 118 | 61 | 6 | 8 | 185 | 123 | -1.495591 | 37.764258 |
| 96 | 113 | 26 | 4 | 0 | 143 | 28.6 | -4.992329 | 36.496704 |
| 2 | 162 | 0 | 4 | 0 | 166 | 73.8 | -2.426042 | 36.88123 |
| 94 | 180 | 5 | 4 | 0 | 189 | 189 | -4.717223 | 36.498944 |
| 51 | 107 | 0 | 2 | 0 | 109 | 54.5 | -8.539876 | 40.799327 |
| 48 | 108 | 0 | 2 | 0 | 110 | 11 | -8.682621 | 39.639743 |
| 39 | 132 | 0 | 2 | 0 | 134 | 134 | -8.700418 | 38.019925 |
| 22 | 116 | 7 | 1 | 0 | 124 | 12.4 | -5.977317 | 37.516563 |
| 95 | 109 | 18 | 1 | 1 | 128 | 10.7 | -4.782858 | 36.506324 |
| 31 | 158 | 2 | 1 | 0 | 161 | 107 | -6.841409 | 37.325561 |
| 115 | 123 | 12 | 0 | 1 | 135 | 67.5 | -3.984785 | 36.738426 |
| 81 | 105 | 10 | 0 | 0 | 115 | 11.5 | -0.188186 | 39.025749 |
| 17 | 103 | 8 | 0 | 0 | 111 | 27.8 | -6.325265 | 37.489774 |
| 84 | 107 | 7 | 0 | 0 | 114 | 19 | -0.515893 | 38.326151 |
| 100 | 221 | 12 | 0 | 0 | 233 | 38.8 | -5.25567 | 36.313535 |
| 72 | 149 | 7 | 0 | 0 | 156 | 208 | 0.026445 | 40.040595 |
| 20 | 107 | 4 | 0 | 0 | 111 | 111 | -5.822164 | 37.683674 |
| 107 | 109 | 4 | 0 | 0 | 113 | 113 | -4.359445 | 36.728748 |
| 45 | 271 | 7 | 0 | 0 | 278 | 556 | -8.737325 | 38.97911 |
| 73 | 103 | 2 | 0 | 0 | 105 | 105 | -0.236844 | 39.736717 |
| 4 | 193 | 2 | 0 | 0 | 195 | 7.22 | -1.830126 | 37.159649 |
| 5 | 128 | 0 | 0 | 0 | 128 | 6.4 | -1.479686 | 37.501581 |
| 10 | 175 | 0 | 0 | 0 | 175 | 35 | -5.703013 | 36.136272 |
| 24 | 108 | 0 | 0 | 0 | 108 | 10.8 | -5.075173 | 37.51608 |
| 25 | 107 | 0 | 0 | 0 | 107 | 71.3 | -5.116692 | 37.528022 |
| 32 | 143 | 0 | 0 | 0 | 143 | 2.86 | -6.843514 | 37.322812 |
| 43 | 102 | 0 | 0 | 0 | 102 | 10.2 | -8.928305 | 38.689513 |
| 47 | 101 | 0 | 0 | 0 | 101 | 10.1 | -9.110552 | 39.366756 |
| 52 | 108 | 0 | 0 | 0 | 108 | 18 | -8.474646 | 41.183247 |
| 53 | 106 | 0 | 0 | 0 | 106 | 70.7 | -8.484793 | 41.476362 |
| 54 | 125 | 0 | 0 | 0 | 125 | 250 | -8.41393 | 41.525855 |
| 55 | 106 | 0 | 0 | 0 | 106 | 106 | -8.658534 | 41.940178 |
| 57 | 104 | 0 | 0 | 0 | 104 | 52 | -8.62292 | 42.092108 |
| 58 | 103 | 0 | 0 | 0 | 103 | 25.8 | -8.658063 | 42.302866 |
| 59 | 103 | 0 | 0 | 0 | 103 | 68.7 | -8.651369 | 42.323445 |
| 62 | 109 | 0 | 0 | 0 | 109 | 18.2 | -8.885356 | 42.808449 |
| 63 | 108 | 0 | 0 | 0 | 108 | 9 | -9.199173 | 43.08555 |
| 66 | 105 | 0 | 0 | 0 | 105 | 1.62 | 0.648884 | 40.768315 |
| 67 | 108 | 0 | 0 | 0 | 108 | 18 | 0.616281 | 40.754134 |
| 68 | 155 | 0 | 0 | 0 | 155 | 103 | 0.635843 | 40.753995 |
| 69 | 112 | 0 | 0 | 0 | 112 | 5.33 | 0.396107 | 40.369172 |
| 70 | 121 | 0 | 0 | 0 | 121 | 4.03 | 0.3961 | 40.369172 |
| 71 | 103 | 0 | 0 | 0 | 103 | 206 | 0.390395 | 40.373482 |
| 82 | 107 | 0 | 0 | 0 | 107 | 3.57 | -0.09768 | 38.561201 |
| 83 | 103 | 0 | 0 | 0 | 103 | 17.2 | -0.119707 | 38.554272 |
| 85 | 105 | 0 | 0 | 0 | 105 | 52.5 | -0.694275 | 37.978093 |
| 86 | 106 | 0 | 0 | 0 | 106 | 35.3 | -0.736478 | 37.915425 |
| 87 | 153 | 0 | 0 | 0 | 153 | 30.6 | -1.590345 | 37.409996 |
| 88 | 108 | 0 | 0 | 0 | 108 | 309 | -3.59129 | 37.17732 |
| 103 | 400 | 0 | 0 | 0 | 400 | 400 | -4.376001 | 36.730619 |
