# Supplementary material for: Widespread male sterility and trioecy in androdioecious Mercurialis annua: Its distribution, genetic basis, and estimates of morph‐specific fitness components
Source: Am J Bot. 2024 Oct 31;111(11):e16429. doi: 10.1002/ajb2.16429 (PMC11584041; doi:10.1002/ajb2.16429)
Supplement: Supplementary file 2 — Appendix S2. The number of individuals of each sexual phenotype and the density of eight Mercurialis annua populations monitored from 2016 to 2018. [file AJB2-111-e16429-s002.docx]

**Appendix S2**. The number of individuals of each sexual phenotype and the density of eight *Mercurialis annua* populations that were monitored from 2016 to 2018.

Pop.: unique for each population

Herma: number of hermaphrodites on the surface surveyed

Female: number of females on the surface surveyed

Male: number of males on the surface surveyed

Total: total number of plants with a sex function on the surface surveyed

Density: density of the surface surveyed

Prop_H: proportion of hermaphrodites on the surface surveyed

Prop_F: proportion of females on the surface surveyed

Prop_M: proportion of males on the surface surveyed

Year: year of the observation

GPS: coordinates of the populations

| Pop. | Herma | Female | Male | Total | Density | Prop_H | Prop_F | Prop_M | Year | GPS |
| --- | --- | --- | --- | --- | --- | --- | --- | --- | --- | --- |
| 133 | 225 | 349 | 434 | 1008 | 42.4 | 0.223 | 0.346 | 0.431 | 2016 | 36.72791,  -3.53112 |
| 145 | 361 | 22 | 108 | 491 | 28 | 0.735 | 0.045 | 0.22 | 2016 | 36.73339,  -3.56282 |
| 138 | 475 | 14 | 60 | 549 | 33.1 | 0.865 | 0.026 | 0.109 | 2016 | 36.75472,  -3.88905 |
| 139 | 206 | 18 | 4 | 228 | 12.3 | 0.904 | 0.079 | 0.018 | 2016 | 36.74499,  -4.04198 |
| 121 | 180 | 31 | 25 | 236 | 34.5 | 0.763 | 0.131 | 0.106 | 2016 | 36.75723,  -4.04596 |
| 140 | 1361 | 95 | 256 | 1712 | 18.7 | 0.795 | 0.055 | 0.15 | 2016 | 36.73672,  -3.95346 |
| 143 | 542 | 132 | 80 | 754 | 18.7 | 0.719 | 0.175 | 0.106 | 2016 | 36.73307,  -3.51005 |
| 146 | 145 | 1 | 13 | 159 | 47.1 | 0.912 | 0.006 | 0.082 | 2016 | 36.74682,  -3.56303 |
| 133 | 704 | 509 | 864 | 2077 | 24.5 | 0.339 | 0.245 | 0.416 | 2017 |  |
| 145 | NA | NA | NA | NA | NA | NA | NA | NA | 2017 |  |
| 138 | 824 | 22 | 73 | 919 | 53.2 | 0.897 | 0.024 | 0.079 | 2017 |  |
| 139 | 204 | 5 | 1 | 210 | 5.83 | 0.971 | 0.024 | 0.005 | 2017 |  |
| 121 | 88 | 1 | 33 | 122 | 16.6 | 0.721 | 0.008 | 0.27 | 2017 |  |
| 140 | 1111 | 42 | 227 | 1380 | 12.5 | 0.805 | 0.03 | 0.164 | 2017 |  |
| 143 | NA | NA | NA | NA | NA | NA | NA | NA | 2017 |  |
| 146 | 408 | 79 | 186 | 673 | 37.4 | 0.606 | 0.117 | 0.276 | 2017 |  |
| 133 | 816 | 316 | 813 | 1945 | 18.5 | 0.42 | 0.162 | 0.418 | 2018 |  |
| 145 | NA | NA | NA | NA | NA | NA | NA | NA | 2018 |  |
| 138 | 540 | 16 | 102 | 658 | 37.4 | 0.821 | 0.024 | 0.155 | 2018 |  |
| 139 | 1235 | 97 | 8 | 1340 | 31.9 | 0.922 | 0.072 | 0.006 | 2018 |  |
| 121 | 318 | 10 | 80 | 408 | 32.6 | 0.779 | 0.025 | 0.196 | 2018 |  |
| 140 | 902 | 37 | 215 | 1154 | 10.4 | 0.782 | 0.032 | 0.186 | 2018 |  |
| 143 | NA | NA | NA | NA | NA | NA | NA | NA | 2018 |  |
| 146 | NA | NA | NA | NA | NA | NA | NA | NA | 2018 |  |
