# Supplementary material for: Widespread male sterility and trioecy in androdioecious Mercurialis annua: Its distribution, genetic basis, and estimates of morph‐specific fitness components
Source: Am J Bot. 2024 Oct 31;111(11):e16429. doi: 10.1002/ajb2.16429 (PMC11584041; doi:10.1002/ajb2.16429)
Supplement: Supplementary file 3 — Appendix S3. The number of males, sterile males, females, and hermaphrodites in the offspring of 64 crosses of Mercurialis annua. [file AJB2-111-e16429-s003.docx]

**Appendix S3**. The number of males, sterile males, females, and hermaphrodites of *Mercurialis annua* in the offspring of 64 crosses.

Mother: ID of the mother plant

Father: ID of the father plant

Cross: type of cross, F × M, H × H, or F × H

F: number of females in the progeny

M: number of males in the progeny

H: number of hermaphrodites in the progeny

N: number of neuters in the progeny

Total: total number of progeny

|  |  |  |  |  |  |  |  |
| --- | --- | --- | --- | --- | --- | --- | --- |
| Mother | Father | Cross | F | H | M | N | Total |
| 10.1 | 24.1 | FxM | 10 | 6 | 9 | 1 | 26 |
| 10.1 | 8.3 | FxM | 13 | 6 | 7 | 0 | 26 |
| 13.2 | 8.3 | FxM | 22 | 11 | 13 | 5 | 51 |
| 14.1 | 6.1 | FxH | 14 | 10 | 6 | 1 | 31 |
| 16.1 | 24.1 | FxM | 15 | 3 | 10 | 1 | 29 |
| 16.1 | 8.2 | FxM | 13 | 8 | 14 | 4 | 39 |
| 17.1 | 8.2 | FxM | 3 | 22 | 8 | 0 | 33 |
| 17.3 | 8.2 | FxM | 16 | 11 | 13 | 1 | 41 |
| 17.6 | 8.3 | FxM | 8 | 11 | 24 | 0 | 43 |
| 2.7 | 24.1 | FxM | 27 | 17 | 4 | 1 | 49 |
| 26.4 | 8.3 | FxM | 17 | 7 | 11 | 1 | 36 |
| 27.6 | 6.1 | FxH | 24 | 27 | 5 | 0 | 56 |
| 4.1 | 8.3 | FxM | 14 | 8 | 13 | 0 | 35 |
| 10.1x8.3 | 27.6x6.1 | FxM | 26 | 5 | 19 | 6 | 56 |
| 14.1x6.1 | 16.1x24.1 | FxM | 30 | 16 | 46 | 2 | 94 |
| 15.5x6.1 | 16.1x24.1 | FxM | 34 | 7 | 30 | 3 | 74 |
| 16.1x24.1 | 26.4x8.3 | FxM | 8 | 8 | 11 | 2 | 29 |
| 2.7x24.1 | 2.7x24.1 | HxH | 13 | 34 | 0 | 0 | 47 |
| 27.6x6.1 | 17.6x8.3 | FxM | 53 | 15 | 40 | 26 | 134 |
| 27.6x6.1 | 4.1x8.3 | FxH | 14 | 24 | 0 | 0 | 38 |
| 13.2 | 6.1 | FxH | 5 | 23 | 0 | 0 | 28 |
| 17.6 | 8.3 | FxM | 17 | 5 | 17 | 0 | 39 |
| 10.1 | 8.3 | FxM | 13 | 6 | 13 | 0 | 32 |
| 14.1 | 6.1 | FxH | 13 | 20 | 0 | 0 | 33 |
| 14.1 | 6.1 | FxH | 38 | 98 | 0 | 0 | 136 |
| 10.1 | 8.3 | FxM | 5 | 10 | 7 | 0 | 22 |
| 4.1 | 8.3 | FxM | 9 | 8 | 13 | 0 | 30 |
| 25.1 | 25.1 | HxH | 12 | 42 | 0 | 0 | 54 |
| 23.11 | 25.1 | FxH | 19 | 31 | 0 | 0 | 50 |
| 17.5 | 25.1 | FxH | 11 | 19 | 0 | 0 | 30 |
| 12.16 | 12.16 | HxH | 5 | 17 | 0 | 0 | 22 |
| 4.3 | 12.16 | FxH | 25 | 56 | 0 | 0 | 81 |
| 22.15 | 12.16 | FxH | 32 | 71 | 0 | 0 | 103 |
| 18.3 | 18.3 | HxH | 23 | 69 | 0 | 0 | 92 |
| 3.17 | 18.3 | FxH | 21 | 47 | 0 | 0 | 68 |
| 11.2 | 18.3 | FxH | 17 | 38 | 0 | 0 | 55 |
| 26.8 | 18.3 | FxH | 13 | 29 | 0 | 0 | 42 |
| 12.1 | 4.5 | FxM | 17 | 10 | 30 | 3 | 60 |
| 7.1 | 4.5 | FxM | 32 | 18 | 45 | 2 | 97 |
| 18.9 | 11.4 | FxM | 12 | 8 | 18 | 0 | 38 |
| 22.3 | 11.4 | FxM | 9 | 5 | 10 | 0 | 24 |
| 17.6 | 11.4 | FxM | 18 | 11 | 23 | 0 | 52 |
| 9.1 | 25.1 | FxM | 26 | 16 | 19 | 0 | 61 |
| 12.4 | 25.1 | FxM | 29 | 14 | 20 | 1 | 64 |
| 23.12 | 25.1 | FxM | 16 | 10 | 20 | 0 | 46 |
| 24.3 | 19.3 | FxM | 12 | 5 | 9 | 0 | 26 |
| 11.2 | 19.3 | FxM | 19 | 9 | 30 | 4 | 62 |
| 15.3 | 19.3 | FxM | 25 | 12 | 33 | 2 | 72 |
| 132.10.1 | 132.24.1 | FxM | 20 | 4 | 26 | 1 | 51 |
| 132.20.1 | 132.8.3 | FxM | 13 | 3 | 14 | 0 | 30 |
| 132.12.5 | 132.8.3 | FxM | 21 | 13 | 29 | 0 | 63 |
| 132.28.1 | 132.8.1 | FxM | 19 | 8 | 23 | 0 | 50 |
| 132.7.8 | 132.8.1 | FxM | 12 | 4 | 20 | 0 | 36 |
| 132.14.8 | 132.8.1 | FxM | 6 | 5 | 14 | 0 | 25 |
| 132.5.17 | 132.17.1 | FxM | 18 | 7 | 23 | 1 | 49 |
| 132.18.3 | 132.17.1 | FxM | 23 | 10 | 30 | 1 | 64 |
| 132.25.9 | 132.17.1 | FxM | 15 | 4 | 20 | 0 | 39 |
| 132.5.3 | 132.5.3 | HxH | 17 | 50 | 0 | 0 | 67 |
| 132.14.1 | 132.5.3 | FxH | 12 | 20 | 0 | 0 | 32 |
| 132.11.8 | 132.5.3 | FxH | 9 | 13 | 0 | 0 | 22 |
| 132.25.3 | 132.5.3 | FxH | 21 | 37 | 0 | 0 | 58 |
| 132.19.1 | 132.19.1 | HxH | 12 | 30 | 0 | 0 | 42 |
| 132.7.4 | 132.19.1 | FxH | 15 | 28 | 0 | 0 | 43 |
| 132.21.2 | 132.19.1 | FxH | 12 | 29 | 0 | 0 | 41 |
