# Supplementary material for: Widespread male sterility and trioecy in androdioecious Mercurialis annua: Its distribution, genetic basis, and estimates of morph‐specific fitness components
Source: Am J Bot. 2024 Oct 31;111(11):e16429. doi: 10.1002/ajb2.16429 (PMC11584041; doi:10.1002/ajb2.16429)
Supplement: Supplementary file 4 — Appendix S4. The number of males, sterile males, females, and hermaphrodites in the offspring of 39 F × M crosses of Mercurialis annua used for recombination rate fitting, and P‐values for fitting each color‐coded scenario (see Figure 5) to the sex ratio of each cross. [file AJB2-111-e16429-s004.docx]

**Appendix S4**. The number of males, sterile males, females, and hermaphrodites of *Mercurialis annua* in the offspring of 39 F × M crosses used for recombination rate fitting, and *P*-values for fitting each color-coded scenario (see Figure 5) to the sex ratio of each cross.

Cross: ID of the cross

Mother: ID of the mother plant

Father: ID of the father plant

F: number of females in the progeny

M: number of males in the progeny

H: number of hermaphrodites in the progeny

N: number of neuters in the progeny

Total: total number of progeny

| Cross | Mother | Father | F | H | M | N | Total | Blue | Red | Green | Black | Orange | Pink |
| --- | --- | --- | --- | --- | --- | --- | --- | --- | --- | --- | --- | --- | --- |
| 1 | 10.1 | 24.1 | 10 | 6 | 9 | 1 | 26 | 0.272 | 0 | 0.444 | 0.005 | 0 | 0 |
| 2 | 10.1 | 8.3 | 13 | 6 | 7 | 0 | 26 | 0.021 | 0 | 0.084 | 0 | 0 | 0 |
| 3 | 13.2 | 8.3 | 22 | 11 | 13 | 5 | 51 | 0.158 | 0 | 0 | 0 | 0 | 0 |
| 4 | 16.1 | 24.1 | 15 | 3 | 10 | 1 | 29 | 0.222 | 0 | 0.126 | 0 | 0 | 0 |
| 5 | 16.1 | 8.2 | 13 | 8 | 14 | 4 | 39 | 0.612 | 0 | 0.011 | 0 | 0 | 0 |
| 6 | 17.1 | 8.2 | 3 | 22 | 8 | 0 | 33 | 0 | 0 | 0 | 0.006 | 0.003 | 0 |
| 7 | 17.3 | 8.2 | 16 | 11 | 13 | 1 | 41 | 0.02 | 0 | 0.136 | 0 | 0 | 0 |
| 8 | 17.6 | 8.3 | 8 | 11 | 24 | 0 | 43 | 0 | 0 | 0.081 | 0.049 | 0 | 0 |
| 9 | 26.4 | 8.3 | 17 | 7 | 11 | 1 | 36 | 0.091 | 0 | 0.147 | 0 | 0 | 0 |
| 10 | 4.1 | 8.3 | 14 | 8 | 13 | 0 | 35 | 0.015 | 0 | 0.375 | 0 | 0 | 0 |
| 11 | 10.1x8.3 | 27.6x6.1 | 26 | 5 | 19 | 6 | 56 | 0.493 | 0 | 0 | 0 | 0 | 0 |
| 12 | 14.1x6.1 | 16.1x24.1 | 30 | 16 | 46 | 2 | 94 | 0 | 0 | 0.964 | 0 | 0 | 0 |
| 13 | 15.5x6.1 | 16.1x24.1 | 34 | 7 | 30 | 3 | 74 | 0.035 | 0 | 0.018 | 0 | 0 | 0 |
| 14 | 16.1x24.1 | 26.4x8.3 | 8 | 8 | 11 | 2 | 29 | 0.161 | 0 | 0.119 | 0.099 | 0 | 0 |
| 15 | 27.6x6.1 | 17.6x8.3 | 53 | 15 | 40 | 26 | 134 | 0.103 | 0 | 0 | 0 | 0 | 0 |
| 16 | 17.6 | 8.3 | 17 | 5 | 17 | 0 | 39 | 0.017 | 0 | 0.4 | 0 | 0 | 0 |
| 17 | 10.1 | 8.3 | 13 | 6 | 13 | 0 | 32 | 0.04 | 0 | 0.624 | 0 | 0 | 0 |
| 18 | 10.1 | 8.3 | 5 | 10 | 7 | 0 | 22 | 0 | 0 | 0.017 | 0.196 | 0.002 | 0 |
| 19 | 4.1 | 8.3 | 9 | 8 | 13 | 0 | 30 | 0.011 | 0 | 0.475 | 0.025 | 0 | 0 |
| 20 | 12.1 | 4.5 | 17 | 10 | 30 | 3 | 60 | 0.045 | 0 | 0.324 | 0 | 0 | 0 |
| 21 | 7.1 | 4.5 | 32 | 18 | 45 | 2 | 97 | 0 | 0 | 0.922 | 0 | 0 | 0 |
| 22 | 18.9 | 11.4 | 12 | 8 | 18 | 0 | 38 | 0.007 | 0 | 0.783 | 0.001 | 0 | 0 |
| 23 | 22.3 | 11.4 | 9 | 5 | 10 | 0 | 24 | 0.093 | 0 | 0.78 | 0.004 | 0 | 0 |
| 24 | 17.6 | 11.4 | 18 | 11 | 23 | 0 | 52 | 0.001 | 0 | 0.584 | 0 | 0 | 0 |
| 25 | 9.1 | 25.1 | 26 | 16 | 19 | 0 | 61 | 0 | 0 | 0.015 | 0 | 0 | 0 |
| 26 | 12.4 | 25.1 | 29 | 14 | 20 | 1 | 64 | 0.002 | 0 | 0.042 | 0 | 0 | 0 |
| 27 | 23.12 | 25.1 | 16 | 10 | 20 | 0 | 46 | 0.003 | 0 | 0.586 | 0 | 0 | 0 |
| 28 | 24.3 | 19.3 | 12 | 5 | 9 | 0 | 26 | 0.066 | 0 | 0.34 | 0 | 0 | 0 |
| 29 | 11.2 | 19.3 | 19 | 9 | 30 | 4 | 62 | 0.158 | 0 | 0.1 | 0 | 0 | 0 |
| 30 | 15.3 | 19.3 | 25 | 12 | 33 | 2 | 72 | 0.014 | 0 | 0.856 | 0 | 0 | 0 |
| 31 | 132.10.1 | 132.24.1 | 20 | 4 | 26 | 1 | 51 | 0.012 | 0 | 0.271 | 0 | 0 | 0 |
| 32 | 132.20.1 | 132.8.3 | 13 | 3 | 14 | 0 | 30 | 0.043 | 0 | 0.413 | 0 | 0 | 0 |
| 33 | 132.12.5 | 132.8.3 | 21 | 13 | 29 | 0 | 63 | 0 | 0 | 0.582 | 0 | 0 | 0 |
| 34 | 132.28.1 | 132.8.1 | 19 | 8 | 23 | 0 | 50 | 0.004 | 0 | 0.674 | 0 | 0 | 0 |
| 35 | 132.7.8 | 132.8.1 | 12 | 4 | 20 | 0 | 36 | 0.008 | 0 | 0.561 | 0 | 0 | 0 |
| 36 | 132.14.8 | 132.8.1 | 6 | 5 | 14 | 0 | 25 | 0.018 | 0 | 0.628 | 0.061 | 0 | 0 |
| 37 | 132.5.17 | 132.17.1 | 18 | 7 | 23 | 1 | 49 | 0.045 | 0 | 0.928 | 0 | 0 | 0 |
| 38 | 132.18.3 | 132.17.1 | 23 | 10 | 30 | 1 | 64 | 0.007 | 0 | 0.954 | 0 | 0 | 0 |
| 39 | 132.25.9 | 132.17.1 | 15 | 4 | 20 | 0 | 39 | 0.009 | 0 | 0.451 | 0 | 0 | 0 |
